# A Survey of *Neotropiella* Handschin, 1942 (Collembola, Neanuridae, Pseudachorutinae) with the Description of a New Brazilian Species

**DOI:** 10.3390/insects11070438

**Published:** 2020-07-13

**Authors:** Bruno C. Bellini, Wanda M. Weiner, Gabriel C. Queiroz, Raiane V. Paz

**Affiliations:** 1 Laboratório de Collembola, Departamento de Botânica e Zoologia, Centro de Biociências, Universidade Federal do Rio Grande do Norte—UFRN. BR 101, Lagoa Nova, Campus Universitário, Natal 59072-970, Brazil; 2 Institute of Systematics and Evolution of Animals, Polish Academy of Sciences, Sławkowska 17, Pl–31–016 Kraków, Poland; weiner@isez.pan.krakow.pl; 3 Museu Nacional, Departamento de Entomologia, Universidade Federal do Rio de Janeiro—UFRJ. Quinta da Boa Vista, São Cristóvão, Rio de Janeiro 20940-040, Brazil; gabriel_cq@yahoo.com.br

**Keywords:** *Aethiopella* Handschin, chaetotaxy, identification key, Neanuroidea, plurichaetosis, review, taxonomy

## Abstract

*Neotropiella* Handschin, 1942 is a pantropical genus of Pseudachorutinae with 18 species, 16 of which are from the Neotropical Region and 13 from Brazil. The genus has several species with unclear descriptions. Herein, we describe a new species of *Neotropiella*, survey the genus based on published papers and discuss its morphology, providing an updated genus diagnosis plus a detailed comparison table and a key to all known species. *Neotropiella arretada* sp. nov. is unique in the combination of a postantennal organ with 14–20 vesicles, its mandible with five teeth, its maxilla apically pointed and its unguis with a pair of reduced teeth. Widely distributed taxa may be species complexes, especially due to their unclear descriptions.

## 1. Introduction

*Neotropiella* Handschin, 1942 was proposed as a subgenus of *Aethiopella* Handschin, 1942, to group species of the genus with five to seven eyes [1]. In 1949, Stach [2] raised *Neotropiella* to genus level, a position maintained by Massoud in his review of the Austral Neanuroidea [3]. After the transfer of *N. malkini* Arlé, 1981 to *Sernatropiella* Palacios-Vargas, 2019, it currently encompasses 18 pantropical species, of which 16 described were from the Neotropical Region [4,5,6,7,8]. The genus is one of the most representative and widespread groups of Pseudachorutinae (Neanuridae) in the Neotropics [6,9]. In Brazil, *Neotropiella* is the most diverse genus of Poduromorpha, with 13 recorded species: *Neotropiella arlei* Najt, Thibaud and Weiner, 1990; *N. barbatae* Queiroz, Silveira and Mendonça, 2013; *N. carli* (Denis, 1924); *N. denisi* (Arlé, 1939); *N. digitomucronata* Thibaud and Massoud, 1983; *N. insularis* Queiroz, Silveira and Mendonça, 2013; *N. macunaimae* Queiroz, Silveira and Mendonça, 2013; *N. meridionalis* (Arlé, 1939); *N. minima* Thibaud and Oliveira, 2010; *N. plurichaetosa* Thibaud and Oliveira, 2010; *N. quinqueoculata* (Denis, 1931); *N. silvestrii* (Denis, 1929); and *N. vanderdrifti* Massoud, 1963 [6,9,10,11,12,13,14,15,16,17,18]. Most Brazilian taxa have been recorded from humid forests or their surroundings within the Atlantic and Amazon forests domains [6,9,12,14,18].

Previous diagnoses of *Neotropiella* separate the genus from other Pseudachorutinae by the combination of the apical bulb of the fourth antennal segment trilobed, the sense rods of the third antennal segment organ within a single integumental cavity, the postantennal organ moruliform (with at least two rings of vesicles), 5 + 5 or 6 + 6 eyes [6], the maxilla styliform without fringed or toothed lamellae, and the furca present with developed dens and mucro [3,6]. Species with complex mandibles (with about eleven to twelve teeth) are the only ones to have 6 + 6 eyes, which raises questions about their identities as *Neotropiella* taxa [6]. Nevertheless, the genus is similar to *Arlesia* Handschin, 1942 in the reduction of eyes, but the latter lacks postantennal organ [1,3]. Also the species with 5 + 5 eyes of *Neotropiella* have large eyes, quite near to each other, while they are smaller and spaced further apart in *Arlesia* [3,6], and the features concerning antennal, body and leg chaetotaxy are dissimilar [7]. *Neotropiella* also resembles *Aethiopella* and *Pseudachorutes* Tullberg, 1871, but differs from both especially in having reduced eyes (8 + 8 in both genera) and a moruliform postantennal organ (a simple ring of vesicles on *Pseudachorutes*) [3,19,20]. The antennal and body chaetotaxy of *Neotropiella* is similar to that of *Pseudachorutes*, as it possesses a microsensillum (**ms**) on Ant. IV, little or no plurichaetosis on head and body, and **M** chaeta between **A** and **B** rows on tibiotarsus. These character states separate *Neotropiella* from the Neotropical genera with derived characteristics, such as the absence of **ms** on Ant. IV, paurochaetotic or plurichaetotic head and body (*Arlesia* and *Handschinurida* Queiroz, 2015 groups, respectively) and basally displaced **M** chaeta on tibiotarsus [7,21].

Herein, we describe a new species of *Neotropiella* from Brazil, update the diagnosis of the genus, provide additions and corrections to the comparative table shown in [6], discuss the genus and provide a key to all known species.

## 2. Materials and Methods

The specimens were collected using pitfall traps during 2018, from forested areas within the Atlantic Forest phytogeographic domain in the Nísia Floresta municipality, Rio Grande do Norte state, Brazil. They were sorted under a stereomicroscope, preserved in 70% ethanol and stored at 6 °C; posteriorly they were diaphanized in Nesbitt’s solution at 50 °C, washed in Arlé’s liquid and mounted on glass slides in Hoyer’s solution [22,23]. Morphology analyses and line drawings were made with drawing tube attached to Leica DM500 and DM750 microscopes (Wetzlar, Germany). Photographs were taken with a Leica MC170HD camera (Wetzlar, Germany) attached to a DM750 microscope using LAS 4.12 software. Final figures were made on CorelDraw X8 software (23 April 2020). The type series is deposited at the Collembola Collection of Centro de Biociências of Universidade Federal do Rio Grande do Norte, Brazil (CC/UFRN), and at the Institute of Systematics and Evolution of Animals, Polish Academy of Sciences, Kraków, Poland (ISEA).

The terminology used to label structures follows: Yosii (1960) with additions of Jordana et al. (1997) to dorsal head and trunk chaetotaxy, with a few adaptations [23,24]. Dorsal thorax I chaetotaxy was only numbered due to its unclear homology as pointed out by Cassagnau [25]. Dorsal thorax II to abdomen IV fields of chaetae are labeled as proposed by Deharveng (1983) and used by Potapov and Banasco (1985), but with merging the dorsolateral (Dl) and lateral (L) fields on a single DL field [26,27]. Ventral abdomen II–V fields of chaetae follow Deharveng (1983) [26]. Labial chaetotaxy follows [3,26,28]. Dorsal sensilla on the fourth and third antennal segments and tibiotarsal chaetotaxy follows [7,26,28].

Abbreviations used in the descriptions are as follows: Abd. = abdominal segment(s); Ant. = antennal segment(s); PAO = postantennal organ; Th. = thoracic segment(s). Chaetae labels are marked in bold.

## 3. Results

### 3.1. Taxonomic Summary and Genus Diagnosis

Order Poduromorpha Börner, 1913 [29]
Superfamily Neanuroidea Massoud, 1967 *sensu* Deharveng, 2004 [3,30]
Family Neanuridae Börner, 1901 [31]
Subfamily Pseudachorutinae Börner, 1906 [32]
Genus *Neotropiella* Handschin, 1942 [1]

*Diagnosis*. Head and body pigmented, mostly dark blue or violet to black; eyepatches black; oral cone, legs and furca generally white to yellowish; distal antenna variably pigmented; body oval, compressed dorso-ventrally; paratergites and paratergal areas variably developed; Ant. III and IV strongly fused, ventral limit seen only in some species; Ant. IV variable in length, shorter, subequal to or longer than Ant. III; Ant. IV mostly with trilobed apical bulb, rarely four-lobed, dorsally with five to seven normal sensilla, microsensillum (**ms**) mostly present, ventrally with or without sensorial field; Ant. III organ composed of two short sensory rods protected by a well-developed cuticular fold, dorsal and ventral guard sensilla (**Sgd** and **Sgv**, respectively) present and subequal, ventral microsensillum (**ms**) present; oral cone elongated; mandible with three to six teeth; maxilla capitulum styliform formed by one to three lamellae entirely fused or divided apically (hooked); PAO moruliform, with at least two rings composed of 7–80 vesicles; 5 + 5 large eyes, close to each other; Th. I with 2 + 2 to 5 + 5 dorsal chaetae, except for *N. murphyi* Massoud, 1964, which has 6 + 6 central chaetae plus several lateral chaetae (plurichaetosis); unguis with one internal tooth, with or without one to two pairs of lateral teeth; unguiculus absent; ventral tube mostly with four chaetae on each side, rarely with three; tenaculum with three teeth on each ramus; furca present and complete, with well-developed dens and mucro, each dens with six chaetae, rarely with five or seven chaetae; anal spines absent (adapted and updated from [3,6,33,34]).

*Type species*. *Neotropiella silvestrii* (Denis, 1929) [16].

*Remarks*. Here we dismiss *N. denisi* and *N. mirabilis* (Handschin, 1929) [33] from *Neotropiella* due to their remarkably complex mandibles, with about 12 teeth and 6 + 6 small eyes, a very different morphology compared to other *Neotropiella* species as previously noted by [6]. Both species should be considered as *incertae sedis* since they cannot be clearly placed in any other genus of Pseudachorutinae for now.

### 3.2. Neotropiella arretada sp. nov. Paz, Bellini, and Queiroz

**Figure 1:**
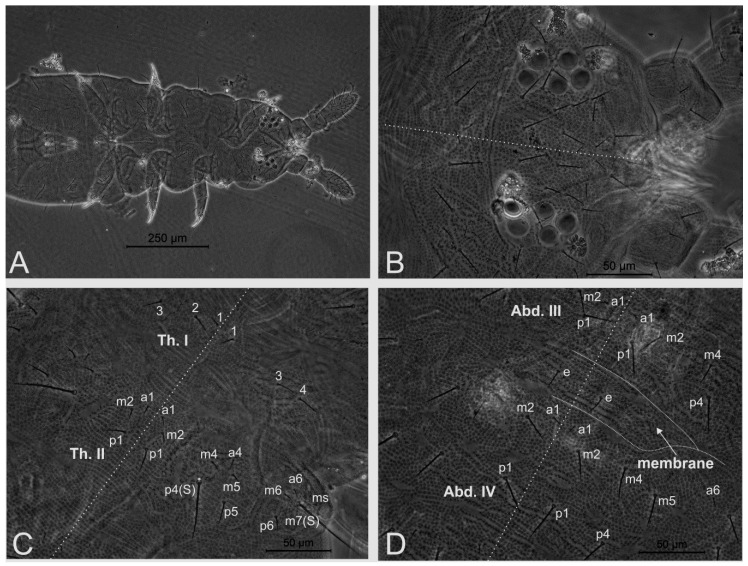
*Neotropiella arretada* sp. nov. holotype: (**A**) partial habitus (Abd. V and VI omitted); (**B**) dorsal head; (**C**) Th. I–II dorsal chaetotaxy (**m2** absent on right side of Th. I, S = long sensilla); (**D**) Abd. III–IV partial dorsal chaetotaxy plus intersegmental membrane (e = extraordinary chaetae). Midline drawn over (**B**–**D**).

**Figure 2:**
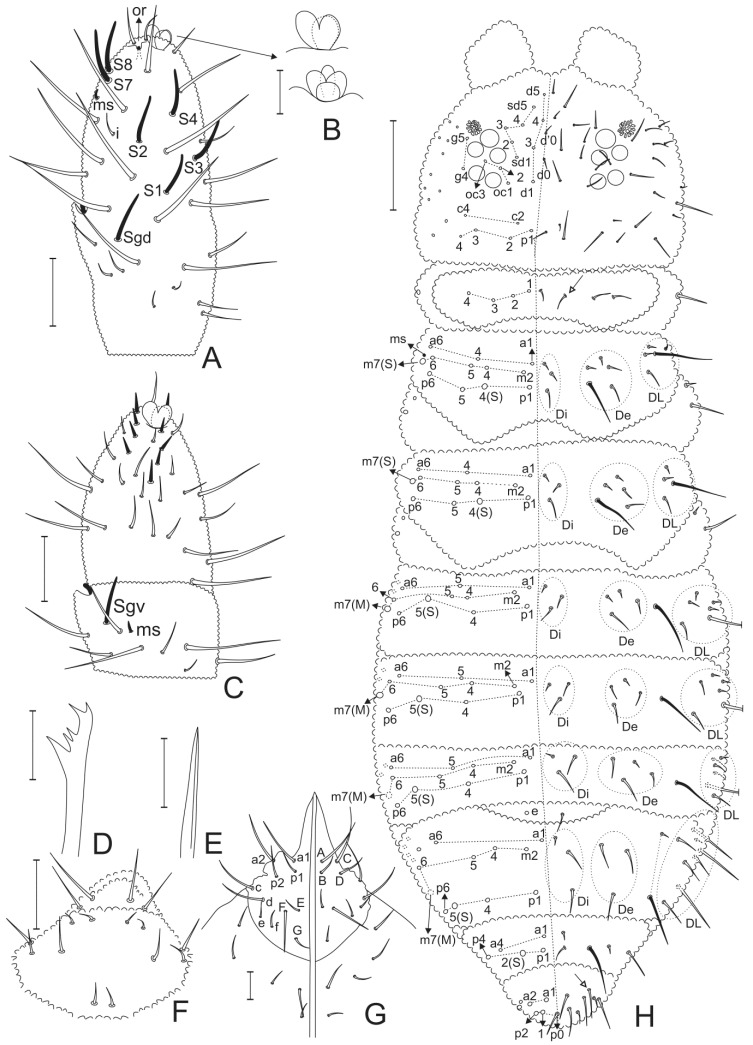
*Neotropiella arretada* sp. nov.: (**A**) left Ant. IV–III, dorsal view; (**B**) detail of different morphologies of Ant. IV apical bulb; (**C**) left Ant. III–IV, ventral view; (**D**) right mandible apex; (**E**) right maxilla capitulum; (**F**) clypeus and labrum morphology; (**G**) labial and postlabial chaetotaxy, left follows [28] and right [3] labial nomenclatures; (**H**) body dorsal chaetotaxy, S = long sensilla, M = macrochaetae (white arrows point to chaetae present or absent). Scale bars: 5 μm (**D**,**E**); 10 μm (**B**); 20 μm (**A**,**C**,**F**,**G**); 100 μm (**H**).

**Figure 3:**
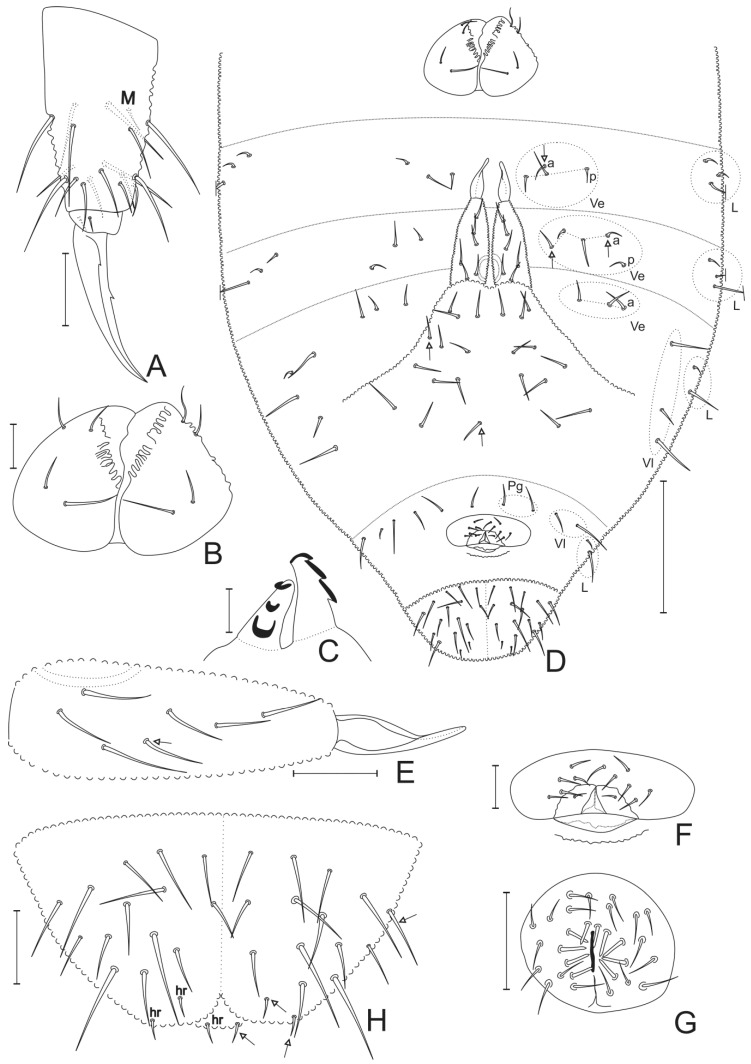
*Neotropiella arretada* sp. nov.: (**A**) tibiotarsus and empodial complex III (inner view); (**B**) ventral tube; (**C**) tenaculum rami and corpus; (**D**) ventral abdominal chaetotaxy, tenaculum omitted; (**E**) right dens and mucro, dorsal view; (**F**) female genital plate; (**G**) male genital plate; (**H**) ventral anal valves and **hr** chaetae on dorsal anal valve. White arrows point to chaetae present or absent. Scale bars: 5 μm (**C**); 10 μm (**F**); 20 μm (**A**,**B**,**E**,**G**,**H**); 100 μm (**D**).

Figure 1, Figure 2 and Figure 3.

*Type material.* Holotype: female on slide (CC/UFRN): Brazil, Rio Grande do Norte State, Nísia Floresta municipality, “Lagoa Redonda” Farm, 06°02′47.5″ S 35°11′42.3″ W, 49 m, 16–18.vi.2018, pitfall trap, Paz, R.V and Carvalho, M.N.A coll. Paratypes (CC/UFRN): two females, one male and four subadults (juveniles) on slides, same data as holotype. Paratype (ISEA): one female on slide, same data as holotype. Paratypes (CC/UFRN): one male and one subadult on slides, same data as holotype, except for sampling date: 13–15.iv.2018.

*Diagnosis.* Ant. IV with three to four lobes on apical bulb, with six (**S1**–**S4**, **S7**–**S8**) dorsal sub-cylindrical sensilla, microsensillum (**ms**) present, ventral sensorial field absent, Ant. IV ventrally with nine broad, blunt chaetae; Ant. III–IV ventral separation marked; Ant. II and I with 11–12 and 7 chaetae, respectively; PAO with 14–20 vesicles; 5 + 5 large eyes; mandible with five teeth; maxilla styliform with two lamellae completely fused, apically pointed; dorsal chaetotaxy mostly composed of primary chaetae (lacking clear plurichaetosis), at least some of dorsal chaetae barbed. Th. I with 4 + 4 dorsal chaetae; tibiotarsus I–III with 19, 19 and 18 chaetae, respectively; ungues with a pair of reduced lateral teeth; ventral tube with 4 + 4 chaetae; manubrium with 10–12 chaetae; dens with six to seven dorsal chaetae; males with 4 + 4 modified chaetae on the genital plate.

*Description.* Color in ethanol dark blue with black eyepatches; distal antennae, oral cone, legs and furca whitish. Habitus oval to elongated. Paratergites reduced (habitus “pseudochorutinian” *sensu* Massoud) [3] (Figure 1A). Body granules medium sized, uniformly distributed on tegument (Figure 1B–D). Body length of holotype: 1.15 mm, range of type series (adults only) 0.88–1.53 mm, females averaging 1.19 mm, males 0.88 mm, adults 1.09 mm.

Head (Figure 1B and Figure 2). Ratio cephalic diagonal: antenna in holotype = 1:1.05, in adult paratypes = 1:1–1.08. Ant. IV with trilobed apical bulb in most specimens, two paratypes with four-lobed apical bulb on only one antenna; dorsally with six subcylindrical sensilla (**S1**–**S4**, **S7**–**S8**), reduced **i** chaeta, dorsolateral microsensillum (**ms**) and subapical organite (**or**) present; ventrally without sensorial field but with nine broad, blunt chaetae (Figure 2A–C). Ant. III and IV dorsally fused, ventral separation marked (Figure 2C). Sensory organ of Ant. III with two small club-shaped sensilla, bent towards each other and protected by cuticular fold, surrounded by two longer and subcylindrical subequal guard sensilla, one dorsal and another ventral (**Sgd** and **Sgv**, respectively), ventral microsensillum (**ms**) present (Figure 2C). Ant. I and II with 7 and 11–12 chaetae, respectively. Mandible with five teeth, one basal and one subapical larger plus three smaller teeth (Figure 2D). Maxilla capitulum styliform, formed by two entirely fused lamellae (lateral one very reduced), apically pointed (Figure 2E). Clypeal area with 6 + 6 and labrum of 2 + 2 chaetae (Figure 2F). Oral cone elongated. Labial palp chaetae **a1**–**a2** (**A** and **C**) longer than others (Figure 2G). Cephalic groove with 2 + 2 surrounding postlabial chaetae (Figure 2G). Eyes 5 + 5, enlarged, anterior eye subequal to or slightly larger than others, eyepatches with three interocular chaetae (**oc1**–**oc3**) (Figure 1B and Figure 2H). PAO moruliform, subequal to or slightly smaller than anterior eye, with 14–20 vesicles (Figure 2H). Dorsal head chaetotaxy (Figure 2H) with pointed micro (average 12 μm in holotype) and slightly clavate mesochaetae (average 25 μm in holotype), **d0** and **d’0** as unpaired chaetae. Dorsal head chaetae slightly barbed.

Trunk dorsal chaetotaxy (Figure 1C,D and Figure 2H). Dorsal thorax and abdomen with heterochaetosis formed by pointed microchaetae (average 8 μm in holotype), slightly clavate mesochaetae (average 22 μm in holotype), slightly clavate macrochaetae (average 37 μm in holotype), and long sensilla (average 54 μm in holotype) (Figure 1C,D and Figure 2H). Most dorsal meso and macrochaetae slightly barbed. Dorsal trunk chaetotaxy mostly composed of primary chaetae (without plurichaetosis). Half trunk sensillar formula from Th. I to Abd. VI as 022/111110 (Figure 2H). Th. I with 4 + 4 chaetae, holotype abnormal with 4 + 3, **1** as microchaeta, **2**–**4** as mesochaetae; Th. II to Abd. IV half trunk dorsointernal (Di)/dorsoexternal (De)/dorsolateral (DL) fields with Di 33/3333, De 55/4443 and DL 54/6668 chaetae, respectively (Figure 1C,D and Figure 2H). Th. II–III chaetotaxy similar, with 3 + 3 chaetae on **a** row (**a1**, **a4** and **a6**), 5 + 5 on **m** row (**m2**, **m4**–**m7**) and 4 + 4 on **p** row (**p1**, **p4**–**p6**); Th. II–III long sensilla as **p4** and **m7**; lateral microsensillum (**ms**) present on Th. II (Figure 1C and Figure 2H). Abd. I–III main chaetotaxy similar, with 3 + 3 chaetae on **a** row (**a1**, **a4** and **a6**), 5 + 5 on **m** row (**m2**, **m4**–**m7**) and 4 + 4 on **p** row (**p1**, **p4**–**p6**), long sensillum as **p5**, **m7** as macrochaeta. A pair of extra chaetae (**e** chaetae) on the membrane between Abd. III and IV (Figure 1D and Figure 2H). Abd. IV main chaetotaxy with 2+2 chaetae on **a** row (**a1**, **a6**), 5 + 5 on **m** row (**m2**, **m4**–**m7**) and 4 + 4 on **p** row (**p1**, **p4**–**p6**), long sensillum as **p5**, **m7** as macrochaeta, **a5** absent. Abd. V main chaetotaxy with 2 + 2 on **a** row (**a1** and **a4**) and 3 + 3 on **p** row (**p1, p2** and **p4**), long sensillum as **p2**. Abd. VI with unpaired mesochaeta **p0** (Figure 2H). Pseudopores not seen, apparently missing.

Trunk appendages and ventral abdomen (Figure 3). Chaetotaxy of legs I–III: Subcoxa I, 1/2/2; subcoxa II, 0/2/2; coxa, 3/8/7; trochanter, 5/5/5; femur, 12/11/10; tibiotarsus, 19/19/18. Tibiotarsus **M** chaeta present, aligned to **B** row or slightly more distal (Figure 3A). Unguis with a single median inner tooth, laterally with a pair of reduced proximal teeth; anterior and posterior pretarsal chaetae present (Figure 3A). Ventral tube with 4 + 4 chaetae (Figure 3B). Tenaculum with three teeth on each ramus (Figure 3C). Abdominal segments I–V ventrally with 0/3–4/3–5/3/4 central chaetae (excluding genital plates) by half body, respectively. Abd. II–IV half body ventral (Ve)/ventrolateral (Vl)/lateral (L) fields with: Ve 3–4(0–1a + 3p)/3–5(2–4a + 1p)/3(3a), Vl 0/0/3, L 3/3/2 chaetae, respectively (Figure 3D). Furca well developed: manubrium with 10–12 chaetae on each side; each dens with six chaetae (one latero-proximal longer than others), except for two paratypes with seven chaetae; mucro tapering at apex, average ratio mucro: dens of holotype = 2.31 (Figure 3D,E). Female genital plate with 1 + 1 smaller eugenital chaetae plus about 11 larger circumgenital chaetae (Figure 3F). Abd. V ventrally with 2+2 pregenital (Pg), 2 + 2 ventrolateral (Vl) and 2 + 2 lateral (L) chaetae (Figure 3D); male genital plate with 5 + 5 thick modified eugenital chaetae plus about 19 circumgenital chaetae (Figure 3G); Abd. V ventrally with 3 + 3 pregenital, 2 + 2 ventrolateral and 2 + 2 lateral chaetae. Paired ventral anal valves with 13–14 ordinary chaetae plus two rear margin (**hr**) microchaetae each (holotype lacking both **hr** microchaetae on right anal valve) (Figure 3D,H); dorsal anal valve with 13–15 ordinary chaetae plus 1 + 1 **hr** microchaetae (holotype lacking 1 **hr** microchaeta on right side) (Figure 2H and Figure 3H).

*Etymology.* “Arretada” or “arretado” is a regional expression commonly used in northeastern Brazil which means “nice”.

*Distribution and Habitat.* Specimens of *Neotropiella arretada* sp. nov. were collected from sand and litter samples near a freshwater lagoon next to a forested area, about 9km from the seashore. The sampled area belongs to the Atlantic Forest phytogeographic domain. It is the same type of locality as the recently described *Aethiopella ricardoi* Paz, Queiroz and Bellini, 2019 [20].

*Remarks. Neotropiella arretada* sp. nov. closely resembles *N. arlei*, *N. barbatae* and *N. minima* in having Ant. IV with six dorsal sensilla plus **ms**, sensorial field absent on ventral side of Ant. IV, Ant I–II with 7 and 11–12 chaetae, respectively, dorsal trunk lacking plurichaetosis, plus tibiotarsi I–III with 19, 19 and 18 chaetae, respectively (unknown to *N. minima*). However, the new species differs from them by having 14–20 vesicles on the PAO (25 or more in *N. arlei* and *N. barbatae*, 7–10 in *N. minima*), five teeth on the mandible (four in *N. arlei* and *N. minima*, six in *N. barbatae*), the apex of the maxilla pointed (hooked in *N. arlei* and *N. barbatae*), and 4 + 4 chaetae on the dorsal Th. I (3 + 3 in *N. arlei* and *N. minima*). Compared to *N. barbatae*, the new species also has one extra chaeta on the De fields from Th. II to Abd. III. Among the above cited species, *Neotropiella arretada* sp. nov. is possibly more closely related to *N. minima*, due to their shared reduction of PAO vesicles. These two species can also be separated by the shape of the ventral modified chaetae on Ant. IV (blunt in the new species vs. somewhat truncate in *N. minima*).

### 3.3. Identification Key* and Distribution** of Neotropiella Handschin, 1942 Species

1. Unguis with two pairs of lateral teeth … 2
  - Unguis with one pair or devoid of lateral teeth … 4
2. PAO with 30 or less vesicles, maxilla apex hooked, unguis teeth normally developed … *N. gordae* Diaz and Najt, 1995; Venezuela
  - PAO with 50 or more vesicles, maxilla apex pointed, unguis teeth strongly developed … 3
3. PAO with 50–65 vesicles, South America distribution … *N. carli* (Denis, 1924)*; Brazil, French Guiana, Guyana, Peru, Venezuela
  - PAO with 65–70 vesicles, Malaysia distribution … *N. murphyi* Massoud, 1964***; Malaysia
4. Dorsal chaetotaxy of plurichaetotic type … *N. plurichaetosa* Thibaud and Oliveira, 2010; Brazil
  - Dorsal chaetotaxy of normal type (mostly composed of primary chaetae) … 5
5. Ant. IV sensorial field present, with 40 or more modified chaetae … 6
  - Ant. IV sensorial field absent, Ant. IV with 20 or less ventral modified chaetae … 9
6. Ant. IV sensorial field with more than 140 modified chaetae, PAO with 38–40 vesicles … *N. insularis* Queiroz, Silveira and Mendonça, 2013; Brazil
  - Ant. IV sensorial field with about 76 or less modified chaetae, PAO with less than 34 or more than 50 vesicles … 7
7. Ant. IV sensorial field with 40 modified chaetae, PAO with 50–60 vesicles, mandible with 5 teeth, maxilla apex hooked … *N. duranti* Diaz and Najt, 1995; Venezuela
  - Ant. IV sensorial field with 46 or more modified chaetae, PAO with 34 or less vesicles, mandible with 4 teeth, maxilla apex pointed … 8
8. Ant. IV sensorial field with 76 modified chaetae, PAO with 34 vesicles, De field with 5 chaetae on Th. II and III … *N. digitomucronata* Thibaud and Massoud, 1983; Brazil, Ecuador, Guadeloupe, Venezuela
  - Ant. IV sensorial field with 46 modified chaetae, PAO with 20–22 vesicles, De field with 4 chaetae on Th. II and III … *N. pedisensilla* Najt, Thibaud and Weiner, 1990; French Guiana
9. Th. I with 4+4 chaetae … 10
  - Th. I with 3+3 or 2+2 chaetae … 12
10. Ant. IV with 7 dorsal sensilla and 7–8 ventral modified chaetae … *N. macunaimae* Queiroz, Silveira and Mendonça, 2013; Brazil
  - Ant. IV with 6 dorsal sensilla and 9 or more ventral modified chaetae … 11
11. Ant. IV with 18–20 ventral modified chaetae, PAO with 27–29 vesicles, mandible with 6 teeth, maxilla apex hooked, De field with 4 chaetae on Th. II and III … *N. barbatae* Queiroz, Silveira and Mendonça, 2013; Brazil
  - Ant. IV with 9 ventral modified chaetae, PAO with 14–20 vesicles, mandible with 5 teeth, maxilla apex pointed, De field with 5 chaetae on Th. II and III … *N. arretada* sp. nov.; Brazil
12. Adult specimens large sized (more than 3 mm), Ant. IV with 9 ventral modified chaetae, PAO with 25–32 vesicles, De field with 4 chaetae on Th. II and III … *N. arlei* Najt, Thibaud and Weiner, 1990; Brazil, French Guiana
  - Adult specimens small to medium sized (2 mm or less), Ant. IV with 8 or less ventral modified chaetae, PAO with 23 or less vesicles, De field with 5 chaetae on Th. II and III … 13
13. Ant. IV with 7 dorsal sensilla, PAO with 18–23 vesicles, mandible with 6 teeth, Th. I with 2 + 2 chaetae, De field with 4 chaetae on Abd. I-III … *N. vanderdrifti* Massoud, 1963; Neotropical
  - Ant. IV with 6 dorsal sensilla, PAO with 7–10 vesicles, mandible with 4 teeth, Th. I with 3 + 3 chaetae, De field with 2–3 chaetae on Abd. I-III … *N. minima* Thibaud and Oliveira, 2010; Brazil
*Neotropiella carli, *N. meridionalis* (Brazil, Cuba), *N. quinqueoculata* (Neotropical) and *N. silvestrii* (Neotropical) have unclear descriptions, and are herein proposed as *species inquirendae* (see Section 4.1). While *N. carli* can be separated from most species due to the presence of two pairs of lateral teeth on its unguis, *N. meridionalis*, *N. quinqueoculata* and *N. silvestrii* are remarkably similar to other species in their few known features, so the last three species were excluded from the key
  **widespread species distributed in six or more countries, including Central and South America, were considered with Neotropical distribution
  ***see discussion topic 4.3

## 4. Discussion

### 4.1. Species Inquirendae

*Neotropiella carli*, *N. meridionalis*, *N. quinqueoculata* and *N. silvestrii* descriptions lack sufficient data to clearly diagnose each one (see Table 1). Such names should be used with caution, especially outside their type localities. Here we propose *N. carli*, *N. meridionalis*, *N. quinqueoculata* and *N. silvestrii* as *species inquirendae*, and these species must be urgently redescribed so their names can be confidently used. *Neotropiella arlei*’s, *N. digitomucronata*’s and *N. vanderdrifti*’s morphologies are better understood, but they need to have more specimens from their type localities revised, since they were described based on only one or two type specimens (Table 1). For these three species at least, the range of the PAO number of vesicles should be used with caution, as the analysis of more specimens could extend it.

### 4.2. Remarks on Neotropiella araguaensis Rapoport, and Maño, 1969

Arlé listed the Brazilian species of Pseudachorutinae, and suggested that *N. araguaensis* from Venezuela could be a synonym of *N. carli*, based on their similar morphology and distribution (both are recorded from the Amazon Forest) [4]. He also suggested that *N. carli* was widely distributed, from northern Brazil and Peru, to Guyana and French Guyana, and so could possibly occur in Venezuela as well (the type locality of *N. araguaensis*) [4,35]. Diaz and Najt [36] officially proposed this synonym, apparently without further analysis or new data regarding *N. araguaensis.* The Mari Mutt and Bellinger’s catalog of Neotropical springtails [37] provided Arlé’s note, and in its supplements [38,39] endorsed his position, probably based on the mentioned synonymy [36]. The two species are similar, but several important features concerning their morphology and chaetotaxy are as yet unknown. Massoud [3] considered *N. carli* to have a trilobed apical bulb on Ant. IV and 65 vesicles in the PAO. The original description of *N. araguaensis* indicated a four-lobed apical bulb on Ant. IV, although in some specimens it appeared that one of the three main lobes is subdivided, and a PAO with 50–57 vesicles [35]. Since *N. carli* is *species inquirenda* (see Section 4.1) and *N. araguaensis* description lacks important data to diagnose it, we believe the available differences do not support the revalidation of *N. araguaensis* at this time, and it will remain as a synonym of *N. carli*. We therefore merged the morphology and distribution of both species under *N. carli* name in the key and in Table 1.

### 4.3. Remarks on Neotropiella murphyi Massoud, 1964

After our new diagnosis of *Neotropiella*, the sole species outside the Neotropical Region is *N. murphyi*, recorded only from Malaysia [8,34]. While this species shares important diagnostic features with other *Neotropiella* taxa, like 5 + 5 large eyes and mandibles with a low number of teeth, among others, it has a unique type of plurichaetosis. *Neotropiella murphy* has a complex thoracic chaetotaxy, with several multiplets not seen even in *N. plurichaetosa*, the sole Neotropical species with confirmed plurichaetosis (see Table 1). Because of its distribution and dissimilar morphology, *N. murphy* may represent another genus arbitrarily included in *Neotropiella*. However, in the absence of further details, including its dorsal chaetotaxy which is insufficiently described by Massoud [34], we would rather keep this species as *Neotropiella* at this time.

### 4.4. On the Widespread Species of Neotropiella

At least three species of Neotropical *Neotropiella* are widely distributed in South and Central America: *N. quinqueoculata*, *N. silvestrii* and *N. vanderdrifti*, while *N. carli* is widely distributed in the Amazon and Atlantic Forest domains [9,37,38,39]. All four species were included in Massoud’s key [3], but there is much information missing from these taxa (see Section 4.1 and Table 1). *Neotropiella quinqueoculata* is possibly the most widespread species of the four, and according to Arlé [4], it has a remarkably variable morphology, especially regarding its PAO. The name *N. quinqueoculata* may conceal a species complex which should be investigated. Because of this, we used Denis’s drawings [15] to provide a reliable number of PAO vesicles for the species (Table 1). The names *N. silvestrii*, *N. vanderdrifti* and *N. carli* may also hide species complexes.

*N. quinqueoculata* cannot be clearly separated from *N. silvestrii* by most of its characters (see Table 1), except for habitus. Massoud pointed to the more “pseudachorutinian” habitus of *N. quinqueoculata* (with reduced paratergites), and to the more “ceratrimerian” habitus of *N. silvestrii* (with large paratergites) [3]. Although such differences can arguably be applied to separate the species, it is clear both need to be revised and redescribed, as stated in Section 4.1.

### 4.5. Homology and Comparative Morphology of Neotropiella Species

*Neotropiella* apparently shows a stable sensillar trunk chaetotaxy; all species with known dorsal chaetotaxy have a 022/111110 half tergum formula, as seen in *N. arlei*, *N. barbatae*, *N. digitomucronata*, *N. insularis*, *N. macunaimae*, *N. minima*, *N. murphyi*, *N. pedisensilla* Najt, Thibaud and Weiner, 1990, *N. plurichaetosa*, *N. vanderdrifti* and *N. arretada* sp. nov. [6,10,13,14,17,34]. Some of these species also possess extra chaetae on Abd. IV, just above **a1**. Massoud’s drawing of *N. vanderdrifti* suggests these extra chaetae are actually on the intersegmental membrane, between Abd. III and Abd. IV ([17] p. 48, Figure 1D), exactly as seen in *N. arretada* sp. nov. (Figure 1D and Figure 2H). The species with known dorsal chaetotaxy mostly have one chaeta on each side, above **a1**, but *N. arlei*, *N. digitomucronata* and *N. murphyi* apparently lack such extra chaetae, and at least *N. plurichaetosa* shows two pairs of them ([14] p. 138, Figure 2C). Most species of *Neotropiella* have only primary chaetotaxy, but at least two of them show plurichaetosis: *N. murphyi* and *N. plurichaetosa*. However, such species differ in their general morphology (as shown in Table 1) and in the nature of their plurichaetosis, as discussed in the Section 4.3. Another possible plurichaetotic species is *N. carli*, which has never had its chaetotaxy properly studied, possibly due to the large size of adult specimens (usually larger than 5 mm). However, it has a high density of chaetae on the antennae, including a well-developed sensorial field on Ant. IV, which may correlate with body plurichaetosis as well [40].

The large eyes of *Neotropiella* species are not clearly homologous compared to the Poduromorpha species with 8 + 8 eyes. The two posterior eyes are quite possibly F–G, since they are below the intraocular row **oc1**–**oc3** (although the proximity of the F eye to the lateral **g4** chaeta suggests it can also be E), while designations for the three anterior eyes are unclear. Due to their proximity to the interocular chaetae, they could be eyes C–D and H, but due to their proximity to the PAO and **sd2**–**3** chaetae, they could also be interpreted as eyes A–C. The two species with 6 + 6 eyes, previously assigned as *Neotropiella*, *N. denisi* and *N. mirabilis*, do not share such enlarged eyes, and their homology is clearer: *N. denisi* has eyes A–C and E–G, and while Handschin’s drawing of *N. mirabilis*’s eyes is incomplete, it shows at least the eyes are clearly not enlarged ([33] p. 17, Figure 1D).

While several species of *Neotropiella* do not have a sensorial field on Ant. IV, most (if not all) do have modified ventral chaetae (see Table 1), which may be homologous to the sensorial field since they arise in the same antennal region. The species of *Neotropiella* with few modified ventral chaetae on Ant. IV are: *N. arlei*, *N. barbatae*, *N. macunaimae*, *N. minima*, *N. plurichaetosa*, *N. vanderdrifti* and *N. arretada* sp. nov. [6,10,14,17]. In the new species, such chaetae are more similar to the ones of *N. arlei* (broad and somehow pointed), and resemble some species in other Neanuroidea genera, such as *Aethiopella*, *Arlesia*, *Pseudachorutella* Stach, 1949 and *Brachystomella* Ågren, 1903 [2,3,20,41,42]. Even so, within *Neotropiella*, the presence or absence of a sensorial field cannot clearly delimit ingroups, since it does not appear to be congruent with other diagnostic features (Table 1).

### 4.6. Notes on Biogeography of Neotropiella

Most species of *Neotropiella* (if not all; see Section 4.3) are from the Neotropical Region, and all Neotropical taxa are found in South America [8,9,37,38,39]. This pattern strongly suggests South America is the center of origin of *Neotropiella*, and therefore the Andean Cordillera may have represented an important biogeographic barrier to the genus speciation and distribution in the Americas. The northern Andes barrier is mostly represented by Paramo province (high cordilleras, above 3000 m) *sensu* Morrone [43], and its uplift started during the late Cretaceous (from about 100–66 mya) [44], but it became conspicuous more recently during the Miocene (23–7 mya) [45]. In this sense, *Neotropiella* may have arisen by at least 7 mya in the forested areas of South America, and its distribution in the Antilles and Central America could have occurred via the Caribbean Sea, similarly to the model proposed by Christiansen and Bellinger (1994) for the Hawaiin colonization of springtails [46]. To support this hypothesis, all species recorded in Mexico and the Antilles are also recorded in South America.

On the other hand, *Neotropiella murphyi*’s dissimilar distribution could possibly be better explained by two hypotheses: (1) It is a Neotropical species not yet recorded in the Neotropical Region, seen in Malaysia due to recent human-mediated dispersal events like soil and plant transportation during the past five centuries. (2) It is not related to the Neotropical taxa, as suggested in Section 4.3.

## 5. Conclusions

*Neotropiella* is a pantropical genus of Pseudachorutinae, with most of its known species being from the Neotropical Region. Several taxa lack properly detailed diagnoses, which compromise the differentiation of some of the species. We gathered further data from the original descriptions and revisions to better delimit them, but at least *N. carli*, *N. meridionalis*, *N. quinqueoculata* and *N. silvestrii* were herein considered *species inquirendae*. The new diagnosis of *Neotropiella* dismisses the species with 6 + 6 eyes and complex mandibles, *N. denisi* and *N. mirabilis*, both of which are in need of redescription. The detailed description of *N. arretada* sp. nov. may be used as a model for future descriptions or revisions of the *Neotropiella* species. Widespread taxa, like *N. quinqueoculata*, *N. silvestrii, N. vanderdrifti* and *N. carli* may consist of several cryptic species. Following our research there are now 17 species of *Neotropiella*, 13 of them from Brazil.

## Figures and Tables

**Table 1:** Main features of *Neotropiella* Handschin, 1942 species.

Species	Number of Type Series Specimens	Size of Type Series Specimens in mm	Ant. IV Sensilla	Ant. IV Sensorial Field	Ant. IV Ventral Modified Chaetae	Ant. I Chaetae	Ant. II Chaetae	PAO Vesicles	Mandible Teeth	Maxilla Apex	Trunk Chaetae	Trunk Plurichaetosis **	Th. I Chaetae (per Side)	Di Chaetae on Th. II-III/Abd. I-III	De Chaetae on Th. II-III/Abd. I-III	Extra Chaetae (e) between Abd. III-IV (per Side)	Tibiotarsus I–III Formula	Unguis Lateral Teeth	Manubrium Chaetae
*arlei* [6,10]	2	3.4	6	−	9	7	11	25–32	4	h	s?	−	3	3/3	4/3	0?	19/19/18	+(2)	?
*barbatae* [6]	3	0.8–1.15	6	−	18–20	7	12	27–29	6	h	b	−	4	3/3	4/3	1	19/19/18	−	9
*carli* [3,6,11,35]	1	4.3–6.0***	?	+	over 100	?	?	50–65	3–4	p	s?	+?	?	?	?	?	18/18/17?	+(4)	?
*digitomucronata* [6,13]	2	0.7	?	+	76	?	?	34	4	p	s?	−	5	3/3	5/2(1)	1	?	+(2)	?
*duranti* [6,36]	6	2.19–2.59	7	+	40	7	11	50–60	5	h	s?	−	?	?	?	0?	18/18/17	+(2)	?
*gordae* [6,36]	12	2.4–3.57	5	+	28–35	10	13	26–30	4	h*	s?	−	?	?	?	?	18/18/17	+(4)	?
*insularis* [6]	20	2.5–3.8	6	+	over 140	9	12	38–40	5	h	s	−	4	3/3	4/3	1	19/19/18	−	15–16
*macunaimae* [6]	15	0.8–2.23	7	−	7–8	7	12	23–27	5	h	b	−	4	3/3	4/3	1	19/19/18	−	9–10
*meridionalis* [3,6,12]	4	1.3–3.2	?	?	?	?	?	29–30	4	p	?	?	?	?	?	?	?	+(2)	?
*minima* [6,14]	11	0.35–0.65	6	−	6–8	7	11–12	7–10	4	p	s?	−	3	3/4	5/3(2)	1?	?	+(2)	?
*murphyi* [3,6,34]	10	up to 6.0	?	+	?	?	?	65–70	4–5	p	s?	+	6m	3/3	9/7(5)	0	18/18/17	+(4)	?
*pedisensilla* [6,10]	3	1.1	6	+	46	7	12	20–22	4	p	s?	−	4	3/3	4/3	0?	19/19/18	+(2)	?
*plurichaetosa* [6,14]	7	0.6–1.2	6	−	5–8	7	11–12	27–36	5–6	p	s?	+	4	5(6)/6	6(7)/7	2	?	+(2)	?
*quinqueoculata* [3,6,15]	3	up to 1.3	?	?	?	?	?	33–54	4	p	s?	?	?	?	?	?	?	+(2)	?
*silvestrii* [3,6,16,47]	5	2.5–3.0	?	+	29	?	?	35–50	4	p	?	?	?	?	?	?	?	+(2)	?
*vanderdrifti* [3,6,17]	1	2.0	7	−	6	?	?	18–23	6	p?	s?	−	2	2(3)/3(2)	5/4	1	18/18/17?	+(2)	?
*arretada* sp. nov.	11	0.88–1.53	6	−	9	7	11–12	14–20	5	p	b	−	4	3/3	5/4	1	19/19/18	+(2)	10–12

Legends: [] species references; (+) present; (–) absent; (?) unknown/doubtful; (p) pointed; (h) hooked; (s) smooth; b (barbed); (m) plus several lateral multiplets; (*) trilamelate; (**) plurichaetosis of *N. murphyi* and *N. plurichaetosa* is apparently not homologous (see Section 4.3); (***) size of type series specimens of *N. araguaensis* were also included, see the discussion.

## References

[1] Handschin E. (1942). Materialen zur Revision de Collembolen. Die Gattung *Ceratrimeria* C.B. sensu Womersley. Ver. Nat..

[2] Stach J. (1949). The Apterygotan Fauna of Poland in Relation to the World-Fauna of This Grup of Insects. Families: Anuridae and Pseudachorutidae.

[3] Massoud Z., Delamare Deboutteville C., Rapoport E.H. (1967). Monographie des Neanuridae, Collemboles Poduromorphes apiéces buccales modifiées. Biologie de l’Amerique Australe.

[4] Arlé R. (1981). Conspecto das espécies brasileiras de Pseudachorutinae, com descrição de uma espécie nova da Colômbia (Insecta, Collembola). Acta Amaz..

[5] Palacios-Vargas J.G. (2019). An extraordinary new genus and species of Pseudachorutinae (Collembola: Neanuridae) from Colombia. Zootaxa.

[6] Queiroz G.C., Silveira T.C., Mendonça M.C. (2013). New species of *Neotropiella* Handschin, (1942) (Collembola: Neanuridae) from Brazil. Soil Org..

[7] Queiroz G.C., Zeppelini D. (2017). Neotropical Pseudachorutinae (Hexapoda: Collembola: Neanuridae): A comparative morphological study with emphasis on endemic taxa. Zool. Anz..

[8] Bellinger P.F., Christiansen K.A., Janssens F. Checklist of the Collembola of the World. http://www.collembola.org.

[9] Zeppelini D., Queiroz G.C., Bellini B.C. Collembola in Catálogo Taxonômico da Fauna do Brasil. PNUD. http://fauna.jbrj.gov.br/fauna/faunadobrasil/379/.

[10] Najt J., Thibaud J.-M., Weiner W. (1990). Collemboles (Insecta) Poduromorphes de Guyane française. Bull. Mus. Natl. Hist. Nat. Paris.

[11] Denis J.R. (1924). Sur les collemboles du Muséum de Paris (1^re^ partie). Ann. Soc. Entomol. Fr..

[12] Arlé R. (1939). Novas espécies de Pseudachorutini (Collembola) do Rio de Janeiro e arredores. Bol. Biol. Nova Ser..

[13] Thibaud J.-M., Massoud Z. (1983). Les Collemboles des Petites Antilles. III.—Neanuridae (Pseudachorutinae). Rev. Ecol. Biol. Sol..

[14] Thibaud J.-M., Oliveira E.P. (2010). Note sur les Collemboles de L’Amazonie Brésilienne II –Neanuridae: Pseudachorutinae ad. p. avec la description de deux espèces nouvelles. Rev. Fr. d’Entomol..

[15] Denis J.R. (1931). Contributo alla conoscenza del “Microgenton” di Costa Rica. II –Collemboles de Costa Rica avec une contribution au species de l’ordre. Boll. Lab. Entomol. Agr..

[16] Denis J.R. (1929). Notes sur les Collemboles récoltés dans ses voyages par la Professeur F. Silvestri (Descriptions d’espèces nouvelles). I—Collemboles d’Extreme–Orient. Boll. Lab. Entomol. Agr..

[17] Massoud Z. (1963). Les collemboles poduromorphes du surinam. Bull. Mus. Natl. Hist. Nat. Paris.

[18] Abrantes E.A., Bellini B.C., Bernardo A.N., Fernandes L.H., Mendonça M.C., Oliveira E.P., Queiroz G.C., Sautter K.D., Silveira T.C., Zeppelini D. (2012). Errata Corrigenda and update for the ‘Synthesis of Brazilian Collembola: An update to the species list.’ Abrantes et al., (2010). Zootaxa, 2388: 1–22. Zootaxa.

[19] Tullberg T. (1871). Förteckning öfver Svenska Podurider. Öfvers. K. VetenskAkad. Förh..

[20] Paz R.V., Queiroz G.C., Bellini B.C. (2019). A new species of *Aethiopella* Handschin, 1942 (Collembola, Poduromorpha, Neanuridae) from Neotropical Region, with comments on the genus. Zootaxa.

[21] Queiroz G.C. (2015). *Handschinurida* nom. nov. (Collembola, Neanuridae), a substitute name for the homonym *Handschinia* Stach, 1949. Zootaxa.

[22] Arlé R., Mendonça C. (1982). Estudo preliminar das espécies de *Dicranocentrus* Schött, 1893, ocorrentes no Parque Nacional da Tijuca, Rio de Janeiro (Collembola). Rev. Bras. Biol..

[23] Jordana R., Arbea J.I., Simón C., Luciáñez M.J. (1997). Fauna Iberica Vol. 8: Collembola, Poduromorpha.

[24] Yosii R. (1960). Studies on the Collembolan genus *Hypogastrura*. Amer. Midland Nat..

[25] Cassagnau P. (1974). Chétotaxie et phylogénie chez les Collemboles Poduromorphes. Pedobiologia.

[26] Deharveng L. (1983). Morphologie évolutive des Collemboles Neanurinae en particulier de la lignée Néanurienne. Trav. Lab. Écobiol. Arthr. Édaph. Toulose.

[27] Potapov M.B., Banasco J. (1985). A new species of springtails from Cuba with comments on the role of chaetotaxy in diagnostic of *Friesea* spp. (Collembola: Neanuridae) (in Russian). Zool. Zhurnal.

[28] D’Haese C.A. (2003). Homology and morphology in Poduromorpha (Hexapoda, Collembola). Eur. J. Entomol..

[29] Börner C. (1913). Die Familien der Collembolen. Zool. Anz..

[30] Deharveng L. (2004). Recent advances in Collembola systematics. Pedobiologia.

[31] Börner C. (1901). Apterygoten-Fauna von Bremen und der Nachbardistrikte. Abh. Nat. Verein Bremen.

[32] Börner C. (1906). Das System der Collembolen—Nebst Beschreibung neuer Collembolen des Hamburger Naturhistorischen Museums. Jahr. Hamburg. Wissen. Anst..

[33] Handschin E. (1929). Collembola from Abyssinia. Trans. Entomol. Soc. London.

[34] Massoud Z. (1964). Um nouveau Collembole Poduromorpe de Malaisie. Rev. Ecol. Biol. Sol.

[35] Rapoport E.H., Maño S. (1969). Colembolos de Venezuela. I. Acta Biol. Venez..

[36] Díaz A., Najt J. (1995). Collemboles (Insecta) des Andes vénézuéliennes. Bull. Mus. Natl. Hist. Nat. Paris.

[37] Mari Mutt J.A., Bellinger P.F. (1990). A Catalog of Neotropical Collembola, Including Nearctic Areas of Mexico. Flora & Fauna Handbook 5.

[38] Mari Mutt J.A., Bellinger P.F. (1996). Supplement to the catalog of the Neotropical Collembola. Caribb. J. Sci..

[39] Mari Mutt J.A., Bellinger P.F., Janssens F. Checklist of the Collembola: Supplement to the Catalog of the Neotropical Collembola. http://www.collembola.org/publicat/neotrcat.htm.

[40] Arlé R. (1966). Collemboles d’Amazonie. I—Poduromorphes nouveaux ou peu connus et notes biologiques sur *Neotropiella carli* (Denis). Bol. Mus. Paraense Emílio Goeldi Belém N. S. Zool..

[41] Ågren H. (1903). Diagnosen einiger neuen Achorutiden aus Schweden (Vorläufige Mittheilungen). Entomol. Tidskrift.

[42] Bellini B.C., Santos N.M.C., Souza P.G.C., Weiner W.M. (2019). Two new species of Brazilian springtails (Hexapoda: Collembola) with comments on Neotropical *Brachystomella* Ågren and *Seira* (*Lepidocyrtinus*) Börner. Insect Syst. Evol..

[43] Morrone J.J. (2014). Biogeographical regionalisation of the Neotropical region. Zootaxa.

[44] Mescua J., Giambiagi L., Ramos V. (2013). Late Cretaceous Uplift in the Malargüe fold-and-thrust belt (35ºS), southern Central Andes of Argentina and Chile. Andean Geol..

[45] Morrone J.J. (2014). Cladistic biogeography of the Neotropical region: Identifying the main events in the diversification of the terrestrial biota. Cladistics.

[46] Christiansen K.A., Bellinger P. (1994). Biogeography of Hawaiian Collembola: The simple principles and complex reality. Orient. Insects.

[47] Massoud Z., Gruia M., Orghidan T., Nuñez-Jiménez A., Botosaneanu L., Decou V., Negrea S., Viña-Bayés N. (1973). Collemboles Arthropléones de Cuba récoltés en 1969 par la mission cubano-roumanie. Résultats des expéditions biospéologiques cubano-roumaines à Cuba.

